# 3D microvascular architecture of pre-cancerous lesions and invasive carcinomas of the colon

**DOI:** 10.1054/bjoc.2001.1809

**Published:** 2001-05

**Authors:** M A Konerding, E Fait, A Gaumann

**Affiliations:** Department of Anatomy and Clinic of General and Abdominal Surgery; Johannes Gutenberg-Universität Mainz

**Keywords:** angiogenesis, tumour vascular architecture, colorectal adenocarcinoma, adenoma, pre-cancerous lesions

## Abstract

Despite the significance of tumour neoangiogenesis and the extensive knowledge on the molecular basis of blood vessel formation currently no quantitative data exist on the 3D microvascular architecture in human primary tumours and their precursor lesions. This prompted us to examine the 3D vascular network of normal colon mucosa, adenomas and invasive carcinomas by means of quantitative microvascular corrosion casting. Fresh hemicolectomy specimens from 20 patients undergoing cancer or polyposis coli surgery were used for corrosion casting, factor VIII and VEGF immunostaining. In addition, immunostaining was done on colorectal tissue from 33 patients with metastatic and non-metastatic carcinomas, polyposis coli and adenomas. This first quantitative analysis of intervessel and interbranching distances, branching angles and vessel diameters in human cancer specimens revealed distinct patterns of the microvascular unit in the tumour centre and periphery. Irrespective of the tumour localization and grading all individual tumours displayed qualitatively and quantitatively the same vascular architecture. This gives further evidence for the existence of a tumour type-specific vascular architecture as recently demonstrated for experimental tumours. Metastatic tumours displayed different vascular architectures only within hot spots, in terms of smaller intervascular distances than in non-metastatic tumours. Pre-cancerous lesions have in part virtually the same vascular architecture like invasive carcinomas. Comparison of VEGF immunostaining also suggests that angiogenesis sets in long before the progress towards invasive phenotypes and that the so-called angiogenic switch is more likely a sequence of events. © 2001 Cancer Research Campaign www.bjcancer.com


*British Journal of Cancer* (2001) **84**(10), 1354–1362
© 2001 Cancer Research Campaign
doi: 10.1054/ bjoc.2001.1809, available online at http://www.idealibrary.com on IDEAL®

http://www.bjcancer.com

### 3D microvascular architecture of pre-cancerous lesions and invasive carcinomas of the colon

**MA Konerding[1], E Fait[1] and A Gaumann[1,2]**

Department of Anatomy[1], and Clinic of General and Abdominal Surgery[2], Johannes Gutenberg-Universität Mainz

**Summary** Despite the significance of tumour neoangiogenesis and the extensive knowledge on the molecular basis of blood vessel formation currently no quantitative data exist on the 3D microvascular architecture in human primary tumours and their precursor lesions. This prompted us to examine the 3D vascular network of normal colon mucosa, adenomas and invasive carcinomas by means of quantitative microvascular corrosion casting. Fresh hemicolectomy specimens from 20 patients undergoing cancer or polyposis coli surgery were used for corrosion casting, factor VIII and VEGF immunostaining. In addition, immunostaining was done on colorectal tissue from 33 patients with metastatic and non-metastatic carcinomas, polyposis coli and adenomas. This first quantitative analysis of intervessel and interbranching distances, branching angles and vessel diameters in human cancer specimens revealed distinct patterns of the microvascular unit in the tumour centre and periphery. Irrespective of the tumour localization and grading all individual tumours displayed qualitatively and quantitatively the same vascular architecture. This gives further evidence for the existence of a tumour type-specific vascular architecture as recently demonstrated for experimental tumours. Metastatic tumours displayed different vascular architectures only within hot spots, in terms of smaller intervascular distances than in non-metastatic tumours. Pre-cancerous lesions have in part virtually the same vascular architecture like invasive carcinomas. Comparison of VEGF immunostaining also suggests that angiogenesis sets in long before the progress towards invasive phenotypes and that the so-called angiogenic switch is more likely a sequence of events. © 2001 Cancer Research Campaign http://www.bjcancer.com

**Keywords**: angiogenesis; tumour vascular architecture; colorectal adenocarcinoma; adenoma; pre-cancerous lesions

There is no doubt that secondary growth of blood vessels, so-called angiogenesis (Jakob et al, 1977), plays an important role in a variety of different pathological conditions (Folkman, 1995). In particular, angiogenesis has been described to be essential for the growth and persistence of solid tumours and their metastases (Folkman et al, 1989; Millauer et al, 1994) and is known to be modulated by a variety of different molecules (Yancopoulos et al, 1998).

A widely accepted concept describes a pre-vascular growth phase of solid tumours which is followed by an angiogenic switch to a vascular phase initiated through the release of angiogenesis factors (Folkman et al, 1989; Carmeliet, 2000). However, there is also evidence that neoangiogenesis sets in before the shift towards invasive phenotypes (Smith-McCune and Weidner, 1994).

In addition, tumour vessel density is in a variety of tumours a predictive parameter for metastatic propagation and/or overall survival or relapse-free survival, e.g., in breast (Weidner et al, 1991, 1992) and prostate cancer (Weidner et al, 1993), in melanomas (Herlyn et al, 1987), colorectal carcinomas (Ishikawa et al, 1999) and oesophageal carcinomas (Kitadai et al, 1998).

We have recently shown that xenografts from different murine and human tumour cell lines display significant differences in their microvascular architecture in terms of tumour type-specific intervascular distances, interbranching distances, as well as vascular diameters (Konerding et al, 1999). This inherent tumour architecture is widely retained even under the influence of highly angiogenic and/or tumourigenic factors such as FGF-II (Konerding et al, 1998).

However, no quantitative data exist on the 3D microvascular architecture in human primary tumours and their precursor lesions. This prompted us to examine the 3D vascular network of normal colon mucosa, adenomas and invasive carcinomas by means of microvascular corrosion casting. Comparisons with the expression of VEGF as an angiogenic factor of utmost importance should elucidate the time course of new vessel formation in the progression from adenomas to invasive carcinomas.

*Received 27 October 2000*
*Revised 22 February 2001*
*Accepted 28 February 2001*

*Correspondence to:* MA Konerding

### MATERIALS AND METHODS

### Patients and specimens

Fresh hemicolectomy specimens were obtained from 19 patients undergoing surgery for colorectal cancer and 1 patient with polyposis coli plus a carcinoma for microvascular corrosion casting and factor VIII and VEGF staining. In 6 surgical specimens between 1 and 6 additional adenomas were found in the normal mucosa distant from the tumours. The patients had a mean age of 66 ± 3 years (12 male, 8 female).

In addition, 14 patients with colorectal cancer and lymph node, liver and/or peritoneal or lung metastases, 12 patients without metastases (13 male, 13 female, mean age: 65 ± 2), 3 patients with polyposis coli (2 male, 1 female, mean age: 51 ± 7 years) and 6

patients with solitary adenomas (4 male, 2 female, mean age: 62 ± 4 years) were examined by factor VIII and VEGF immunostaining only.

The specimens studied were staged according to the TNM classification of the UICC (5th edition 1997) and the Dukes classification as follows: 12 patients with villous adenomas; 4 patients with polyposis coli, 25 patients with non-metastatic tumours (1 Dukes A, $T_1$, $N_0$, $M_0$; 24 Dukes B, $T_{2–3}$, $N_0$, $M_0$) and 21 patients with metastatic tumours (5 Dukes C, $T_x$, $N_{1–2}$, $M_0$, 16 Dukes D $T_x$, $N_x$, $M_1$). The grading was in most cases G2 (41 patients G2, 5 patients G3).

All surgical specimens and tissue samples were obtained from the Department of General and Abdominal Surgery of the Johannes Gutenberg-University Mainz with the written consent of the patients in accordance with the local ethical committee's approval and the regulations laid down by law.

### Microvascular corrosion casting

The specimens were transferred immediately after surgery to the Department of Anatomy. The main supplying arteries were cannulated with olive-tipped cannulas. Blood was washed out by infusion of body-warm buffered physiological saline added with heparin. Before injection of 80–120 ml Mercox CL-2B (Vilene Med Co, Tokyo Japan) diluted with 20% methylmethacrylate monomers (Merck Darmstadt, Germany), up to 100 ml of 2.5% body temperature glutaraldehyde was perfused as fixative. On average 3–5 tissue blocks with a diameter of 0.8–1.0 cm were excised from the tumour centre, the periphery and the invasive edge and/or polyps. From each specimen at least 2 samples of normal mucosa were harvested distant from the lesion.

After additional tissue excision for immunostaining, the surgical specimens were fixed in 4% formalin and brought to the Department of Pathology. Extensive preceding experiments, which were carried out before this study on human surgical specimens, have shown that the casting procedure does not interfere with the histopathological diagnosis.

The casting specimens were prepared for scanning electron microscopy as described in detail earlier (Konerding et al, 1999). From all specimens between 10 and 25 stereo pairs of peripheral and central areas were recorded as stereo images using a tilt angle of 6˚.

### 3D morphometry

Pairs of stereo images were analysed after 3D reconstruction with an image analysis program (KS 300, Carl Zeiss Vision, Eching, Germany) to calculate parameters that describe the microvascular network architecture such as the intervessel and the interbranching distances as well as the branching angles and diameters. For details of the reconstruction and calculation see Malkusch et al (1995).

### Immunohistology

Primary polyclonal rabbit anti-human factor VIII (DAKO, Hamburg), and anti-VEGF (Zymed Laboratories, USA) antibodies were used. Briefly, the sections were irradiated in a microwave oven, incubated with goat serum and then incubated with the primary antibodies. The secondary biotinylated goat anti-rabbit antibody was connected with either the avidin-biotin-peroxidase complex (Vectastatin ABC-Kit, Vector Laboratories) or with the Envision® kit (Dako, Hamburg, Germany). Finally, the AEC substrate was applied, and the specimens were stained with haematoxylin.

The specimens were analysed blindly by 3 different observers. The extent of the immunostaining for VEGF was estimated by counting the number of positive cells/10 high power fields (diameter 1.235 mm) in a Zeiss axiophot microscope (Zeiss Oberkochen, Germany). Results were classified according to 5 categories: 0, negative; 1, 1–25% cells positive; 2, 26–50% cells positive; 3, 51–75% cells positive; 4, 76–100% cells positive.

In factor VIII staining, in up to 5 different sections per case 1 to 6 hot spots per section were analysed. 5 tumours were excluded due to a poor factor VIII staining. Within the hot spots the vascular densities were evaluated based on the criteria of Weidner et al (1991) by means of an image analysis program (KS 300, Carl Zeiss Vision, Eching, Germany).

### Statistical analysis

Statistical analyses and graphic displays were performed using SigmaStat and SigmaPlot (SPSS, Erkrath, Germany). All groups were tested for the form of their frequency and distribution before analysis. The differences in the frequency distribution were tested for significance using the $\chi^2$ test, whereas the testing for significant differences using the mean and standard error of mean (SEM) of the distribution was performed with the Mann–Whitney rank sum test and analysis of variance (ANOVA). The sensitivity was assessed by means of a power calculation which revealed d values higher than 80% at the 5% level of confidence.

### RESULTS

### Vascular architecture in normal and pathological colorectal mucosa

The mucosal capillary plexus of the large intestine is typically arranged in a regular, hexagonal honeycomb pattern around the mucosal glands (Figure 1A, B). The plexus is supplied by arteries that divide within the submucosa to subepithelial capillaries. Venous drainage takes place by venules originating immediately under the mucosal surface and leading to submucosal veins. This basic pattern is seen in all parts of the large intestine from the caecum to the rectum. However, there is some heterogeneity in the number of capillary layers around each crypt in the individual colonic segments as well as in individual specimens.

The unique branching pattern of the mucosal plexus is not retained in tumours. Low-power magnification of the corrosion casts reveal the characteristic features of tumour vascularity in all specimens examined such as a low or missing vessel hierarchy, heterogeneous intratumour vascular densities, and blind-ending vessels (Figure 1C, D). In general, the vascular density declines from the tumour periphery to the tumour centre. Especially, in these areas with lower vascular density numerous vessel compressions and elongated vessel segments are seen. The vessel diameters in general are increased. Changes in diameter within individual vessel segments suggest altered blood flow characteristics. Comparisons of tumours grown in different colonic segments

© 2001 Cancer Research Campaign

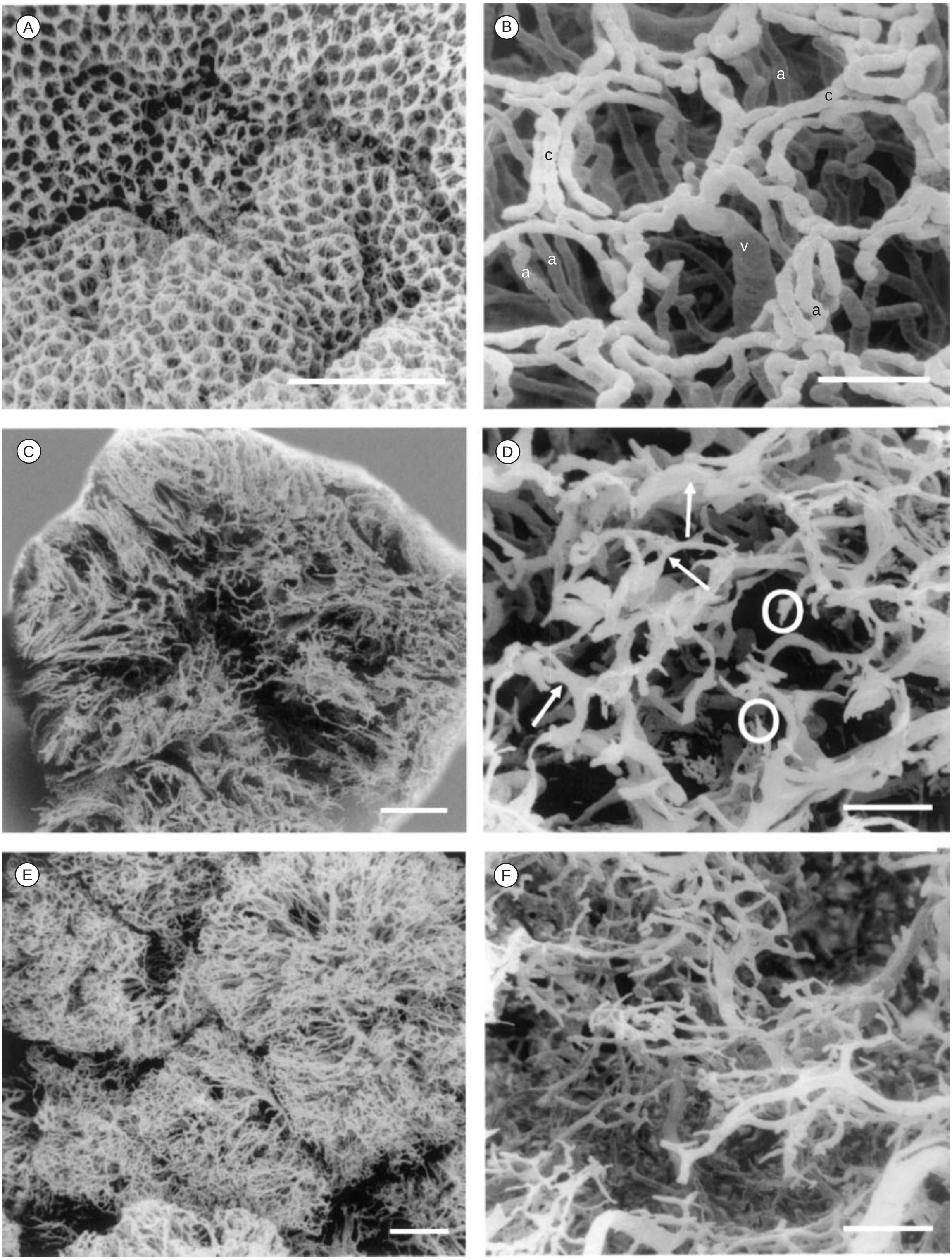

**Figure 1** Scanning electron micrographs of corrosion casts of the colorectal vascularity in normal mucosa (**A, B**), carcinoma (**C, D**) and adenoma (**E, F**). **A, B** In the normal mucosa, the capillaries (**c**) are arranged honeycomb like around the crypts. Ascending arterioles (a) from the submucosa ascending feed this plexus, which is drained by parallel descending veins (v). The overview in **A** shows how the mucosal plexus follows the mucosal foldings. **C, D** Vascular architecture in adenocacinomas. **C**: note the markedly expressed heterogeneity in vascular densities and nearly avascular areas in the centre (adenocarcinoma of descending colon; pT3, pN0, pMx, G2). Vessel compressions, changes in diameter (arrows), and blind ending vessels (○) are seen in all carcinomas (sigmoidal adenocarcinoma; pT3, pN3, pM1, G2). **E**, **F** Solitary adenoma with high vascular density in the luminal surface (**E**) and vascular plexuses in the centre (**F**), again with loss of vascular hierarchy. Bars in **A, C, E** = 1 mm, bars in **B, D, F** = 100 μm did not reveal any obvious differences in the vascular architecture. The same was true for differences in tumour sizes.

The loss of the normal vascular architecture pattern is already visible in solitary adenomas (Figure 1E, F). The reduction of the

*© 2001 Cancer Research Campaign*

vascular hierarchy in the central parts was so pronounced that in 4 of 10 adenomas the examiners were not able to distinguish adenomas from carcinomas when double-blinded. In 6 of 10 adenomas nearly all characteristics of the tumour vascular architecture can be found, however, to varying extents and less expressed than in the carcinomas.

### Morphometry of the microvascular unit in normal colorectal mucosa and carcinomas

Because of the obvious qualitative differences in the peripheral and central tumour parts we analysed the microvessel architecture separately in the tumour centre, the luminal surface and periphery.

The distribution curve of intervessel distances (Figure 2A) as parameter for vascular density of the control mucosa has an uniform shape with a peak at 101 µm. It is plotted on a logarithmic scale because the data most closely approximate to a log normal distribution. The higher vascular densities in the periphery of the tumours shift the curve to the left. The curves of the tumour centre and the luminal tumour surface are flattened and wide based reflecting the heterogeneity in intervessel distances. All differences are highly significant ($P < 0.0001$). Mean values with maximal and minimal values are summarized in Table 1.

Figure 2B shows the quantification of the distances between successive branches. The normal tissue network shows a branching every 40–80 microns. In the peripheral tumour surface nearly identical interbranch distances are seen, whereas in the central tumour surface and tumour centre there are clear differences in the range and the median values and the patterns of the distributions. Branching occurs less frequently and much more heterogeneous when compared to the tumour periphery and the normal mucosa ($P < 0.001$).

The branching angles of the vessels (Figure 2C) range from almost zero to almost 180° with a median value of about 87° in the normal tissue and 72° in the tumours. This is the only parameter which we have analysed that shows no significant differences between the individual tumour areas. However, the distribution curves with smaller angles are significantly different from the normal mucosa ($P < 0.0001$).

When plotting the vessel diameters, distinct patterns emerge for the tumour areas. In all regions, the tumour vessels have significantly wider diameters ($P < 0.0001$). The largest calibre vessels are found in the centre, where also the highest dispersion is seen. Within individual vessel segments, i.e., between 2 successive branches, the diameters vary in the normal tissue less than 5%, whereas in the tumour centre and luminal surface very large variabilities of up to 125% deviation from the mean diameter are seen ($P < 0.0001$; data not shown).

These data prove that the tumour microvascular network differs in many aspects from the normal tissue microvascular architecture but shows also significant differences within the tumour. We have, however, not seen significant differences between individual colorectal tumours or between different tumour localization with the examined parameters. This gives further evidence for the existence of a tumour type-specific vascular architecture.

**Table 1**

| | Intervessel distances | | | |
|---|---|---|---|---|
| | **mean ± SEM** | **minimum** | **maximum** | **number** |
| control | 101.5 ± 0.7 | 41.7 | 181.2 | 1127 |
| tumour periphery | 54.3 ± 0.7 | 3.9 | 494.6 | 2486 |
| luminal tumour surface | 118.6 ± 2.0 | 8.5 | 1181.5 | 2627 |
| tumour centre | 177.7 ± 3.4 | 19.3 | 1756.3 | 2087 |
| adenomas | 53.99 ± 1.0 | 11.5 | 225.23 | 923 |
| polyposis | 92.47 ± 1.3 | 3.88 | 1181.5 | 210 |
| | **Interbranch distances (µm)** | | | |
| | **mean ± SEM** | **minimum** | **maximum** | **number** |
| control | 51.2 ± 0.5 | 2.2 | 238.5 | 3001 |
| tumour periphery | 50.2 ± 0.49 | 5.7 | 336.3 | 3517 |
| luminal tumour surface | 78.5 ± 1.05 | 6.6 | 920.8 | 3467 |
| tumour centre | 131.9 ± 2.39 | 9.18 | 896.1 | 1855 |
| | **Branching angles (∘)** | | | |
| | **mean ± SEM** | **minimum** | **maximum** | **number** |
| control | 87,1 ± 0.7 | 7.4 | 167.9 | 1806 |
| tumour periphery | 72.8 ± 0.69 | 0.57 | 175.6 | 2236 |
| luminal tumour surface | 71.7 ± 0.68 | 1.2 | 171.5 | 2306 |
| tumour centre | 75.9 ± 1.65 | 1.7 | 174.2 | 1742 |
| | **Vessel diameter** | | | |
| | **mean ± SEM** | **minimum** | **maximum** | **number** |
| control | 12 ± 0.1 | 6.4 | 20.9 | 4750 |
| tumour periphery | 19.4 ± 0.1 | 5.6 | 53.5 | 4500 |
| luminal tumour surface | 18.3 ± 0.1 | 2.2 | 84.5 | 4500 |
| tumour centre | 30.9 ± 0.4 | 0.9 | 161.9 | 4750 |

© 2001 Cancer Research Campaign 

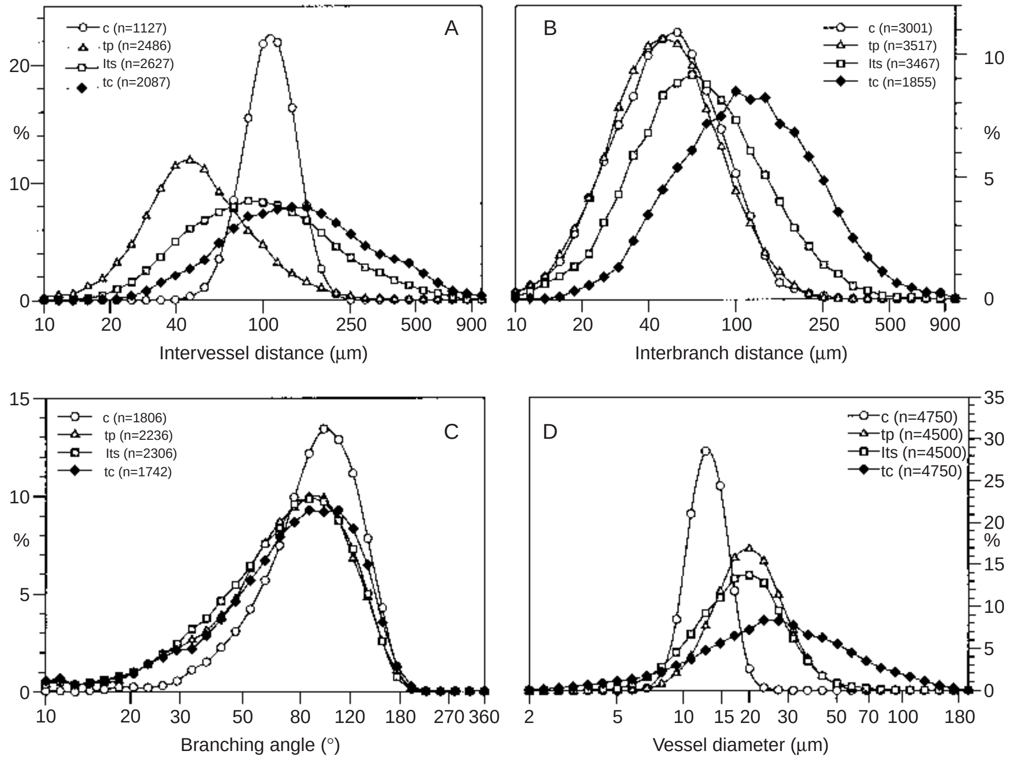

**Figure 2** Common logarithmic distribution of the intervessel distances (**A**), interbranch distances (**B**), branching angles (**C**), and vessel diameters (**D**) quantified in 3D scanning electron micrographs of corrosion casts of 20 colorectal adenocarcinomas and control mucosa. c = control mucosa, tp = tumour periphery, lts = luminal tumour surface, tc = tumour centre. For means, minimum and maximum values see Table 1

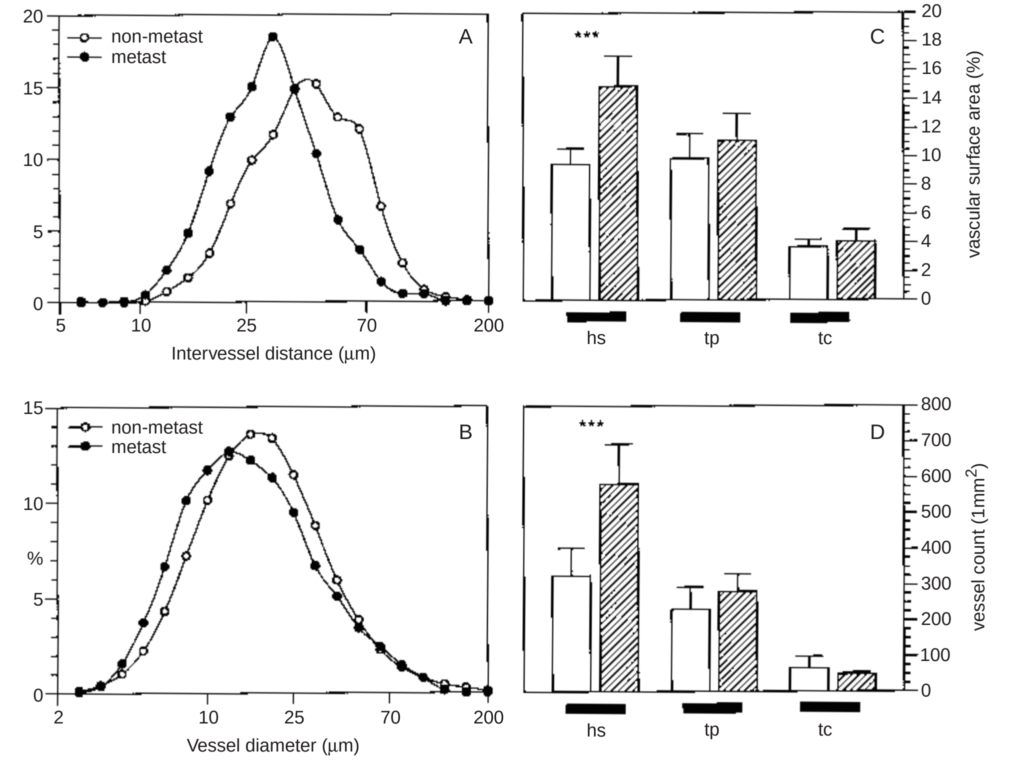

**Figure 3** Intervessel distances (**A**), vessel diameters (**B**), vascular surface volumes (**C**) and vessel counts (**D**) in metastastatic (filled dots and columns) vs. non-metastatic (open dots and columns) colorectal adenocarcinomas. **A** and **B** were measured in 3D reconstructed corrosion casts in areas of highest vascular densities (hot spots), whereas the data shown in **C** and **D** derive from anti-factor VIII stained sections. The obvious difference in intervessel distances in hot spots (**A**; $P < 0.0001$) is paralleled by significant differences ($P < 0.0001$) in the vascular surface areas (**C**) and vessel counts (**D**) within hot spots (hs), whereas in the tumour periphery (tp) and the tumour centre (tc) no significant differences are seen

*© 2001 Cancer Research Campaign*

### Vascular architecture in metastastatic vs. non-metastastatic tumours

Comparisons of primary tumours which have already metastasized with colorectal tumours without metastases at the time of surgery revealed no difference in the vascular architecture in the tumour centre and periphery (data not shown). However, separate measurements of intervessel distance only in the areas of highest vascular density, so-called hot spots, showed significant differences ($P < 0.001$). Figure 3A shows that the intervessel distances in metastatic tumours are shorter than in non-metastatic colorectal adenocarcinomas. The diameters of vessels of metastatic carcinomas are slightly smaller than that of non-metastatic primaries (Figure 3B).

This finding in corrosion casts was cross-checked in a larger series in serial sections stained with anti-factor VIII. In total, 46 patients were examined. Figure 3C and D show that indeed no significant differences can be seen in the vascular densities (vessel count; 3D) or percentage vascular surface areas (3C) in the tumour centre or periphery. In contrast, when measuring these parameters only in the hot spots, a significant result ($P < 0.0001$) is evident.

### Vascularity of pre-cancerous lesions

The adenomas shared many architectural features with the carcinomas despite the better maintenance of the vascular hierarchy. Figure 4A shows that the distribution curve of the intervessel distances has nearly the same shape, median value, minimum and maximum like that of the tumour peripheral surface. However, the variability between individual adenomas is by far higher than between individual carcinomas.

In the case of the patient with polyposis coli a different vascular architecture was observed (Figure 4B). The distribution curve of the intervascular distances showed two distinct peaks. The right peak was congruent with that of the normal mucosa whereas the left peak is comparable to that of adenomas and carcinomas. Obviously, these peaks reflect the progress from normal tissue to malignant lesion.

### VEGF expression in adenomas and carcinomas

Figure 5 shows a comparison of VEGF expression in controls (A), carcinomas (B) and adenomas (C) with virtually no difference between carcinomas and adenomas. All colon carcinomas expressed VEGF detected by immunohistochemistry with a strong cytoplasmic signal. No staining was obtained in normal mucosa adjacent to the tumour areas as well as at the resection margins.

VEGF expression did not differ between adenomas and polyposis coli patients ($P = 0.943$). Similar results were obtained comparing metastatic and non-metastatic adenocarcinomas ($P = 0.566$). However, adenomas and polyposis coli differed significantly from metastatic adenocarcinomas ($P = 0.005$ for adenomas; $P = 0.003$ for polyposis) and non-metastatic carcinomas ($P = 0.024$ for adenomas; $P = 0.012$ for polyposis). The results of the semi-quantitative scoring are summarized in Figure 6.

### DISCUSSION

The significance of angiogenesis for tumour growth and metastasis has been stressed in numerous studies of the structure and biological properties of the tumour vasculature and blood flow (Folkman, 1976; Jain, 1988; Folkman and D'Amore, 1996). Despite the importance of angiogenesis in tumour biology and antiangiogenic and vascular targeting concepts, literature reviews show a divergence of opinions on the question of tumour specificity of the vascular architecture, even though all normal tissues and organs develop a specific and characteristic vascular architecture fulfilling their specific functional and nutritive requirements (for review, see Konerding et al (1995)).

Nearly all studies of the tumour vascular architecture are based on qualitative, descriptive light microscopic and/or angiographic 2D methods, which enable a morphometric assessment of the gross network but cannot adequately describe the 3D microvascular architecture. This can best be done with 3D reconstruction of corrosion casts (Konerding et al, 1995) since the whole terminal blood stream from the arterial to the venous segment including the capillary bed can be clearly shown.

Using this method we have recently demonstrated tumour type-specific vascular architectures in xenografted murine and human tumours (Konerding et al, 1999). Furthermore, the vascular architecture of an experimental tumour does not depend on the size and/or the rate of growth of the lesion but rather on features characteristic of the tumour cell type. In the first attempt to define the

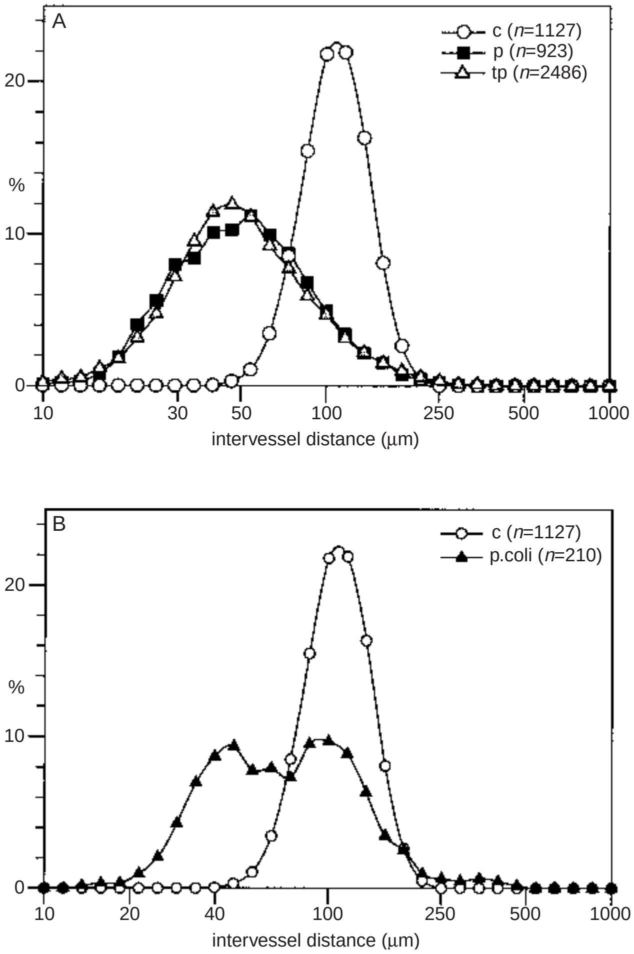

**Figure 4** Common logarithmic distribution of intervessel distances in control colorectal mucosa (c), tumour periphery (tp), and in adenomas (**A**). Note the striking similarity of the vessel densities in carcinomas and adenomas. In **B**, the intervessel distance distribution within the mucosa of polyposis coli (p. coli) reveals a double peak reflecting high vascular densities like in adenomas or tumours, whereas the second peak reflects the normal intervessel distance distribution

*© 2001 Cancer Research Campaign*

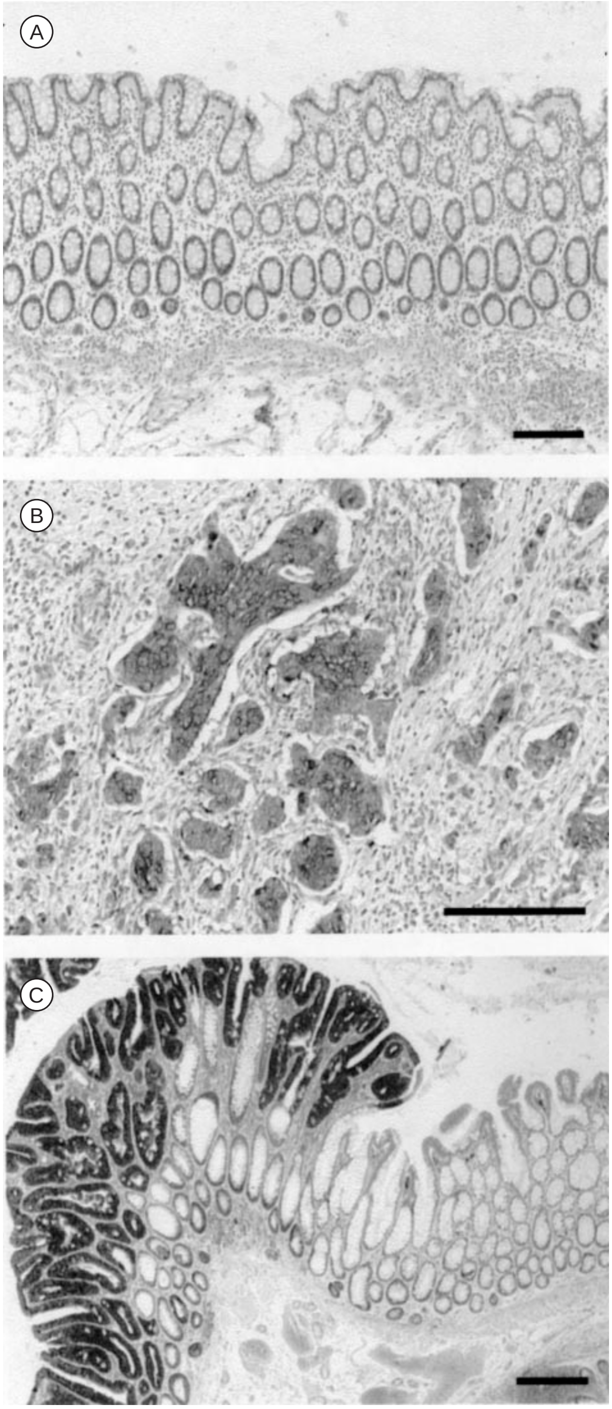

**Figure 5** Expression of VEGF in normal colonic mucosa (**A**), in an adenocarcinoma (**B**), adenocarcinoma of descending colon, pT3, pN1, pMx; G2) and an adenoma (**C**). In **B**, more than 75% of the cells in the tumour tissue express a strong signal, which can also be seen in the adenoma influence of a single, defined growth factor on the 3D tumour vascular pattern, we demonstrated that FGF-2 expression and release induce a faster formation of the microvessel network but do not change the basic geometry (Konerding et al, 1998).

In the present study, our first question was whether human primary colorectal tumours develop a uniform microvascular architecture. A prerequisite for this study was to exclude any differences in the normal mucosa of the individual colorectal segments, since the pre-existing normal vasculature might also determine the tumour vascular network. In a recent corrosion casting study of all colorectal segments, no significant inter- and intra-individual differences of the microvascular architecture were detected (Fait et al, in press). Thus, it was permissible to pool the control data. The present study did not show any significant differences in the microvascular architecture between the individual tumours, which indicates again a tumour type-specific vascularity. However, clear differences were seen in the individual tumour areas, again irrespective of localization and total tumour size. Interbranch and intervessel distances were significantly shorter in the periphery including the invasion front than in the tumour centre. This was also demonstrated in the anti-factor-VIII vessel counts.

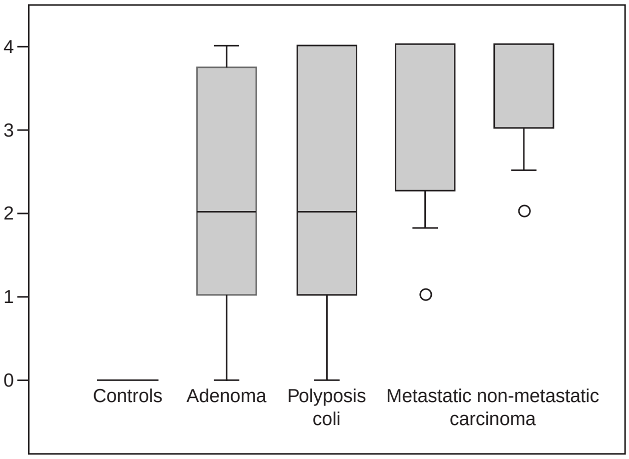

**Figure 6** Semiquantitative scoring of the VEGF expression in control tissue, adenomas, polyposis coli, metastatic and non-metastatic colorectal carcinomas (O, negative; 1, 1–25%; 2, 26–50%; 3, 51–75%; 4, 76–100% of cells positive). Note the high variability of VEGF expression especially in the pre-cancerous lesions as compared to the carcinomas

Vessel counts are increasingly done because of their possible significance as predictive assay. In colonic carcinomas there is strong evidence for their predictive value (Weidner et al, 1993; Takahashi et al, 1997; Wong et al, 1999). At first glance, this seems to be contrary to our finding of a uniform microvascular architecture in colorectal carcinomas. In fact there is no contradiction since we assessed our parameters in at least 10 areas per tumour region covering approximately 30–35% of the tumour volume whereas hot spots make up at best 1% of the tumour volume. When assessing separately the areas of highest vascular densities in our casts, it also turned out that metastatic tumours have a significant lower intervessel distance than non-metastatic tumours. For the parameters interbranch distance, branching angle and vessel diameter, no significant differences were seen. Also our anti-factor VIII immunostaining showed no differences in the vascular surface areas and vessel counts in the tumour centre and in the peripheral areas. Only the hot spots within the periphery and the invading edge were significantly different. Therefore we conclude that the basic microvascular architecture of colorectal carcinomas is retained despite the increase in vessel density within the hot spots of metastatic tumours.

The second question of this paper was the vascularity of precursor lesions. The concept of an ‘angiogenic switch’ with an

 *© 2001 Cancer Research Campaign*

avascular, dormant phase of tumour growth followed by the vascular phase, is widely accepted. Even though numerous details of the molecular biological basis are still unknown, there is strong evidence that the induction of vessel growth may occur early in the adenoma–carcinoma sequence. K-ras mutations, a frequent mutation in colorectal tumours, have been found already in 24% of human colorectal adenomas (Ajiki et al, 1995). Since mutant ras is known to up-regulate VEGF (Arbiser et al, 1997) it seems to be obvious that angiogenesis sets in long before the development of invasive carcinoma. In numerous adenomas we have seen VEGF expression comparable to that in carcinomas supporting this opinion. The difference between adenomas and carcinomas in our series was significant, whereas Wong et al (1999) and Takahashi et al (1998) found no difference. This contradiction may in part be explained by different methodology. On the other hand it is possible that VEGF alone is not sufficient for tumorigenicity of human colorectal carcinoma cells (Okada et al, 1998). Since other growth factors are also involved in angiogenesis processes (Carmeliet, 2000) it is likely that besides VEGF other molecules are needed to effectively promote tumour angiogenesis.

The up-regulation of VEGF in the adenomas obviously induces the described changes in vascular architecture which was very similar to that of the tumour periphery. From that we conclude that a part of the events that are summarized under 'tumour angiogenesis' and the 'angiogenic switch' may take place already before the transformation to the carcinoma and is likely to reflect a continuous process.

A sequence of events is also suggested by the striking similarities of the 3D vascular architecture in a part of the adenomas and carcinomas. We cannot conclusively answer the question in general whether the distinct tumour vascular architecture is correlated to the expression of VEGF. However, morphometry of intervascular distances in polyposis coli specimens, which have shown a high variability in VEGF expression within individual patients, displays a double-humped distribution curve with a first peak congruent with that of the normal mucosa and a second one comparable to that of adenomas and carcinomas in corrosion casts. Therefore, we conclude that most likely only the amount of new vessel formation is correlated to the VEGF expression whereas the overall 3D vascular architecture seems to be determined by the tumour cells. Comparisons of adenocarcinomas expressing lower levels of VEGF had virtually the same microvascular network patterns like that with high VEGF expression levels. This parallels our recent studies on the impact of FGF-2 on the microvascular architecture, where we have shown that the overall new vessel production is positively correlated to the FGF-2 level whereas the basic geometry of the tumour *inherent* vascular network is expressed independent from the FGF-2 level (Konerding et al, 1998).

### ACKNOWLEDGEMENTS

This study was supported by a grant of the Deutsche Forschungsgemeinschaft (Ko 1050/6–1). E Fait was supported by a postgraduate fellowship of the Robert-Müller-Stiftung für Herz-Kreislaufforschung, A Gaumann by a grant of the Deutsche Forschungsgemeinschaft to MK.

We thank Kerstin Bahr for excellent technical support and photographic artwork, Wolf Malkusch, Carl-Zeiss-Vision Germany, for his morphometric expertise and Dr D Thompson,University of Aberdeen, for the critical review of the manuscript.

### REFERENCES

Ajiki T, Fujimori T, Ikehara H, Saitoh Y and Maeda S (1995) K-ras gene mutation related to histological atypias in human colorectal adenomas. *Biotech Histochem* **70**: 90–94

Arbiser JL, Moses MA, Fernandez CA, Ghiso N, Cao Y, Klauber N, Frank D, Brownlee M, Flynn E, Parangi S, Byers HR and Folkman J (1997) Oncogenic H-ras stimulates tumor angiogenesis by two distinct pathways. *Proc Natl Acad Sci USA* **94**: 861–866

Carmeliet P (2000) Mechanisms of angiogenesis and arteriogenesis. *Nat Med* **6**: 389–395

Fait E, Gnoth SH, Dimitropoulou C, Gaumann A, Kirkpatrick CJ, Junginger T and Konerding MA. Microvascular patterns of the human large intestine: Morphometric studies of vascular parameters in corrosion casts. *Scanning Microsc*. In press

Folkman J (1976) The vascularization of tumors. *Sci Am* **234**: 58–63

Folkman J (1995) Angiogenesis in cancer, vascular, rheumatoid and other disease. *Nat Med* **1**: 27–31

Folkman J and D'Amore PA (1996) Blood vessel formation: what is its molecular basis? (comment). *Cell* **87**: 1153–1155

Folkman J, Watson K, Ingber D and Hanahan D (1989) Induction of angiogenesis during the transition from hyperplasia to neoplasia. *Nature* **339**: 58–61

Herlyn M, Clark WH, Rodeck U, Mancianti ML, Jambrosic J and Koprowski H (1987) Biology of tumor progression in human melanocytes. *Lab Invest* **56**: 461–474

Ishikawa H, Fujii H, Yamamoto K, Morita T, Hata M, Koyama F, Terauchi S, Sugimori S, Kobayashi T, Enomoto H, Yoshikawa S, Nishikawa T and Nakano H (1999) Tumor angiogenesis predicts recurrence with normal serum carcinoembryonic antigen in advanced rectal carcinoma patients. *Surg Today* **29**: 983–991

Jain RK (1988) Determinants of tumor blood flow: a review. *Cancer Res* **48**: 2641–2658

Jakob W, Jentzsch KD, Mauersberger B and Oehme P (1977) Demonstration of angiogenesis-activity in the corpus luteum of cattle. *Exp Pathol (Jena)* **13**: 231–236

Kitadai Y, Haruma K, Tokutomi T, Tanaka S, Sumii K, Carvalho M, Kuwabara M, Yoshida K, Hirai T, Kajiyama G and Tahara E (1998) Significance of vessel count and vascular endothelial growth factor in human esophageal carcinomas. *Clin Cancer Res* **4**: 2195–2200

Konerding MA, Miodonski AJ and Lametschwandtner A (1995) Microvascular corrosion casting in the study of tumor vascularity: a review. *Scanning Microsc* **9**: 1233–1243

Konerding MA, Fait E, Dimitropoulou C, Malkusch W, Ferri C, Giavazzi R, Coltrini D and Presta M (1998) Impact of fibroblast growth factor-2 on tumor microvascular architecture. A tridimensional morphometric study. *Am J Pathol* **152**: 1607–1616

Konerding MA, Malkusch W, Klapthor B, van Ackern C, Fait E, Hill SA, Parkins C, Chaplin DJ, Presta M and Denekamp J (1999) Evidence for characteristic vascular patterns in solid tumours: quantitative studies using corrosion casts. *Br J Cancer* **80**: 724–732

Malkusch W, Konerding MA, Klapthor B and Bruch J (1995) A simple and accurate method for 3-D measurements in microcorrosion casts illustrated with tumour vascularization. *Anal Cell Pathol* **9**: 69–81

Millauer B, Shawver LK, Plate KH, Risau W and Ullrich A (1994) Glioblastoma growth inhibited in vivo by a dominant-negative Flk-1 mutant. *Nature* **367**: 576–579

Okada F, Rak JW, Croix BS, Lieubeau B, Kaya M, Roncari L, Shirasawa S, Sasazuki T and Kerbel RS (1998) Impact of oncogenes in tumor angiogenesis: mutant K-ras up-regulation of vascular endothelial growth factor/vascular permeability factor is necessary, but not sufficient for tumorigenicity of human colorectal carcinoma cells. *Proc Natl Acad Sci USA* **95**: 3609–3614

Smith-McCune KK and Weidner N (1994) Demonstration and characterization of the angiogenic properties of cervical dysplasia. *Cancer Res* **54**: 800–804

Takahashi Y, Tucker SL, Kitadai Y, Koura AN, Bucana CD, Cleary KR and Ellis LM (1997) Vessel counts and expression of vascular endothelial growth factor as prognostic factors in node-negative colon cancer. *Arch Surg* **132**: 541–546

Takahashi Y, Bucana CD, Cleary KR and Ellis LM (1998) p53, vessel count, and vascular endothelial growth factor expression in human colon cancer. *Int J Cancer* **79**: 34–38

*© 2001 Cancer Research Campaign* 

Weidner N, Semple JP, Welch WR and Folkman J (1991) Tumor angiogenesis and metastasis-correlation in invasive breast carcinoma. *N Engl J Med* **324**: 1–8
Weidner N, Folkman J, Pozza F, Bevilacqua P, Allred EN, Moore DH, Meli S and Gasparini G (1992) Tumor angiogenesis: a new significant and independent prognostic indicator in early-stage breast carcinoma (see comments). *J Natl Cancer Inst* **84**: 1875–1887
Weidner N, Carroll PR, Flax J, Blumenfeld W and Folkman J (1993) Tumor angiogenesis correlates with metastasis in invasive prostate carcinoma. *Am J Pathol* **143**: 401–409
Wong MP, Cheung N, Yuen ST, Leung SY and Chung LP (1999) Vascular endothelial growth factor is up-regulated in the early pre-malignant stage of colorectal tumour progression. *Int J Cancer* **81**: 845–850
Yancopoulos GD, Klagsbrun M and Folkman J (1998) Vasculogenesis, angiogenesis, and growth factors: ephrins enter the fray at the border (comment). *Cell* **93**: 661–664

 *© 2001 Cancer Research Campaign*
